# Polyamines at the crossroad between cell metabolism and epigenetic regulation in acute leukemias

**DOI:** 10.1038/s41420-025-02573-y

**Published:** 2025-07-02

**Authors:** Francesca Pirini, Anna Ferrari, Mouna Jandoubi, Irene Azzali, Davide Angeli, Rossana Mondrone, Chiara Bracci, Francesca Ruggieri, Giovanni Martinelli, Giorgia Simonetti

**Affiliations:** 1 Biosciences Laboratory, IRCCS Istituto Romagnolo per lo Studio dei Tumori (IRST) “Dino Amadori”, Meldola (FC), Italy; 2 Biostatistics and Clinical Trials Unit, IRCCS Istituto Romagnolo per lo Studio dei Tumori (IRST) “Dino Amadori”, Meldola (FC), Italy; 3 Department of Medical and Surgical Sciences (DIMEC), University of Bologna, Bologna, Italy

**Keywords:** Cancer metabolism, Targeted therapies

## Abstract

Polyamines, namely putrescine, spermidine and spermine, are involved in multiple molecular pathways through their ability to bind nucleic acids and modulate protein stability. Their intracellular level is regulated through biosynthesis, catabolism and uptake from the extracellular milieu and the disruption of their homeostasis contributes to a variety of human disorders including cancer, as mainly described in solid tumors. Recently, there is an increasing interest in understanding polyamine functions in acute leukemias, due to the linkage between leukemic gene drivers, polyamine metabolism alterations and epigenetic defects. In particular, polyamine involvement in the regulation of acetylation and methylation is clinically relevant since epigenetic drugs are currently the backbone of novel therapeutic combinations, especially in acute myeloid leukemia (AML). With the exception of *methylthioadenosine phosphorylase* (*MTAP*), the enzyme leading to methionine regeneration that is frequently deleted in acute lymphoblastic leukemia (ALL), genes involved in polyamine metabolism and the interconnected methionine and arginine pathways are rarely targets of genetic lesions in acute leukemias. Conversely, functional alterations, including elevated polyamine levels and deregulated activity of enzymes involved in their metabolism, have been recently reported in leukemic cells. Notably, the polyamine catabolic enzyme spermidine/spermine N1 acetyltransferase (SAT1) that is overexpressed in AML and associated with a myeloproliferative phenotype, is a tumor suppressor gene in ALL, suggesting diverse mechanisms of action across hematological malignancies according to the lineage commitment and the differentiation stage. In light of the promising results achieved in AML and ALL by selective targeting of protein arginine methyltransferase 5 (PRMT5) and methionine adenosyltransferase 2A (MAT2A), two enzymes at the crossroad between polyamine metabolism and protein methylation, in this review we examine and discuss the role of polyamines in epigenetic regulation and other biological processes supporting leukemic cell survival, proliferation and differentiation, which provides the opportunity to discover additional polyamine-related targets and design novel therapeutic combinations.

## Facts

- Intracellular levels of polyamines and related metabolites are functionally altered in acute leukemias.
- Polyamines regulate the epigenome that is also a therapeutic target in acute leukemias.
- Polyamines interact with leukemogenic transcriptional program regulating EIF5A, TFEB, insulin receptor and NRF2 activity.
- MAT2A and PRMT5 are novel promising therapeutic targets at the crossroad between methionine/polyamine metabolism and epigenetic regulation.

## Open questions

- What is the functional role of polyamines in leukemic cells during resistance to therapy?
- How do polyamines interact with AML driver alterations (e.g. *NPM1* mutations)?
- How do polyamines regulate the epigenome in combined treatments?
- Do combination therapies including inhibitors of polyamine metabolism and its related pathways provide a clinical utility?

## Introduction

Polyamines are organic compounds with two or more amino groups. The three main natural polyamines, putrescine, spermidine and spermine, can be found in all living organisms. Polyamines are characterized by a low molecular mass and a high positive charge, which secure them the ability to bind DNA, RNA and proteins. They are involved in many fundamental cellular processes including gene expression, cell proliferation, differentiation and death, with specificities related to the cell type and its physiological condition [1].

**Fig. 1:**
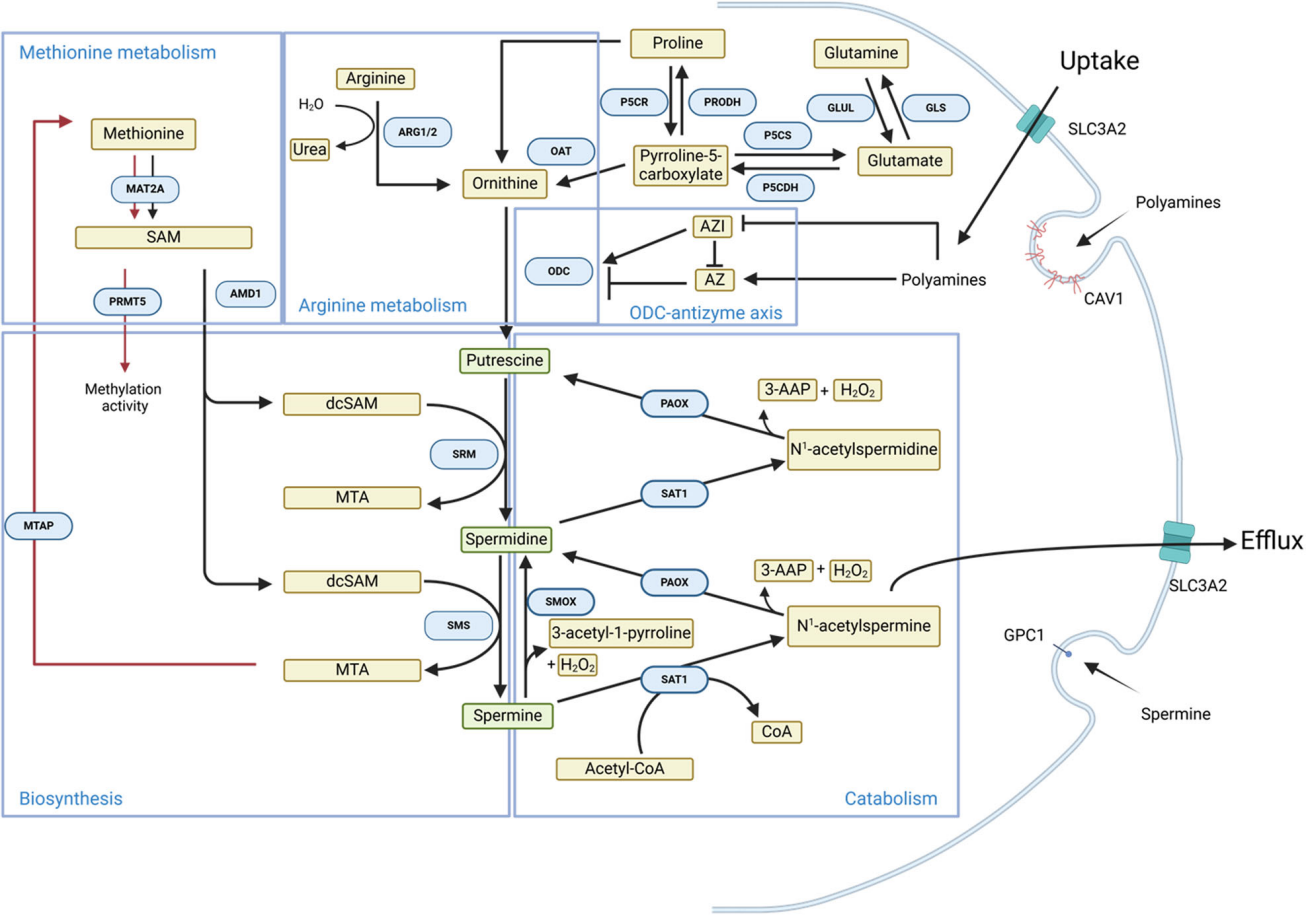
The polyamine metabolic pathway and its connections with methionine and arginine metabolism.

The intracellular level of polyamines is regulated through biosynthesis, catabolism and uptake from the extracellular milieu (Fig. 1). They are produced from ornithine that can be synthesized from arginine, glutamate or proline, and is transformed into putrescine by ornithine decarboxylase (ODC) [2]. Putrescine generates spermidine and spermine by a two-step addition of two aminopropyl groups (left by decarboxylated S-adenosyl-L-methionine, dcSAM) catalyzed by spermidine synthase (SRM) and spermine synthase (SMS), respectively [3]. dcSAM derives from the decarboxylation, mediated by adenosylmethionine decarboxylase 1 (AMD1), of SAM [4], which is produced in the methionine cycle by methionine adenosyltransferase (MAT) enzymes (MAT2A subunit in hematopoietic cells) [5] and also serves as methyl donor to the type I protein arginine methyltransferase 5 (PRMT5) and to a variety of DNA, RNA and protein substrates. Spermidine and spermine synthesis releases 5′-methylthioadenosine (MTA), which is then converted back to methionine and adenosine precursors by methylthioadenosine phosphorylase (MTAP) [6]*. S*permidine oxidase (SMOX), spermidine/spermine N1-acetyltransferase (SAT1) and peroxisomal N1-acetylpolyamine oxidase (PAOX) enzymes are responsible for spermine and spermidine catabolic processes [7].

An altered polyamine metabolism has been detected in a variety of cancer types, mainly solid ones. However, polyamines modulate a number of biological processes that are also involved in malignant transformation, disease progression and/or response to treatment in acute leukemias. In this review we describe polyamine alterations in acute myeloid leukemia (AML) and acute lymphoblastic leukemia (ALL) and provide evidence on their role in epigenetic regulation and other biological processes supporting cell survival, proliferation and differentiation, in order to discuss the therapeutic actionability of the pathway in these malignancies.

## Polyamine alterations in acute leukemias

Intracellular polyamine alterations in AML and ALL were firstly reported about ten years ago, when increased levels of spermidine and spermine, correlating with the blast percentage, were measured in patients’ peripheral blood mononuclear cells compared with healthy individuals, along with elevated SAT1 activity [8]. In ALL, high putrescine intracellular concentration and ODC activity were also observed [8]. Additional studies provided evidence of polyamine metabolism alterations in acute leukemias, resulting from functional deregulation rather than genetic defects in the majority of cases.

### Structural alterations of genes involved in polyamine metabolism

Genes involved in polyamine metabolism (*ODC1*, *SRM*, *SMS*, *SAT1*, *PAOX*, *SMOX*) and in the strictly connected pathways [*AMD1*, *arginase* (*ARG)1/2*, *MAT2A*, *MTAP*, *protein arginine methyltransferase 5* (*PRMT5*), *ornithine aminotransferase* (*OAT*)] are rarely mutated in acute leukemias. Two *ODC1* and one *SMOX* mutations were identified across 1214 AML from the TCGA-LAML, Beat AML, AML TARGET (https://www.cbioportal.org/) and our NGS-PTL (EGAD00001007940, EGAD00001007941) [9, 10] cohorts, and 153 B-ALL and 72 ALL (not otherwise specified) from the St. Jude and ALL TARGET datasets (https://www.cbioportal.org/). *ODC1* mutations include the nonsense M315delinsIIG* (in ALL) and the missense K342T alterations (in AML), that are localized in the pyridoxal-dependent decarboxylase C-terminal sheet protein domain and classified as variants of uncertain significance. One AML patient carried the *SMOX*^G31D^ mutation, defined as variants of uncertain significance but predicted to alter the protein function.

**Fig. 2:**
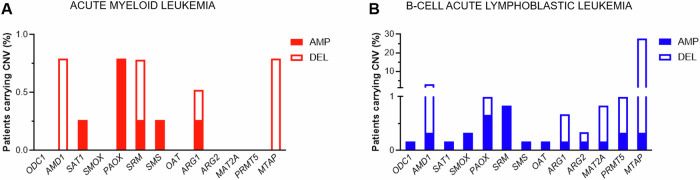
Copy number variation (CNV) of polyamine metabolism-related genes in acute leukemias. Data of AML (**A**) and B-ALL (**B**) patients were retrieved from the TCGA-LAML, AML and ALL TARGET cohorts (https://www.cbioportal.org/).

Copy number alterations (CNAs) of polyamine-related genes are also rare events. They were detected in less than 3% of patients (except for the *MTAP* gene) among 382 AML (Fig. 2A) and 603 B-ALL (Fig. 2B) from the TCGA-LAML, AML and ALL TARGET cohorts, respectively. Genes involved in catabolic reactions, as *SAT1* and *PAOX*, are preferentially amplified. Conversely, in B-ALL, genes related to methionine metabolism (*MAT2A*, *AMD1*, *PRMT5* and *MTAP*) are generally deleted. The genomic regions encoding polyamine-related genes (chromosomes 1, 2, 6, 10, 14, 20 and X) are rarely affected by cytogenetic alterations in AML, suggesting that the reported CNAs may result from a selective pressure conferring a fitness advantage to the malignant cells. For example, *SAT1* amplification may fulfill a heightened need for polyamine acetylated derivatives. Conversely, in B-ALL, chromosomes 6, 10, 14 and X are frequently gained (chromosome 20 has also been reported, with a lower frequency) in high hyperdiploid cases [11], while loss of chromosomes 2 and 20, among others, has been observed in hypodiploid B-ALL [11, 12]. Therefore, some of the observed CNAs may be bystander events resulting from the overall B-ALL karyotype complexity.

An exception is represented by *MTAP* deletion, that occurs in 11–14% and 28–33% of B-ALL [13, 14] and T-ALL [14, 15], respectively, and in 27.4% of cases from the pediatric B-ALL TARGET cohort (Fig. 2B). The *MTAP* gene is located at the 9p21.3 locus, which is a deletion hotspot in ALL [16], as well as in solid tumors, with a high prevalence in pancreatic (18.4%), biliary tract (15.6%), and lung (14.3%) cancers [17]. In Philadelphia-positive (Ph+) B-ALL adult patients *MTAP* is co-deleted with the *cyclin dependent kinase inhibitor (CDKN)2A* gene in 71.4% of cases with 9p21 loss [18]. In T-ALL patients *MTAP* deletion has been associated with poor overall survival [15] and frequently co-occurred with *CDKN2A/2B* deletion [19].

### Functional alterations of polyamine metabolism in acute leukemias

At functional level, 38% of T-ALL (in agreement with the genetic data), 6% of B-ALL, 16.7% of mixed lineage acute leukemia and 7.1% of AML cases did not show any MTAP activity [20]. The data on AML patients reinforce the hypothesis that non-genetic mechanisms drive polyamine metabolism alterations in leukemic cells.

**Fig. 3:**
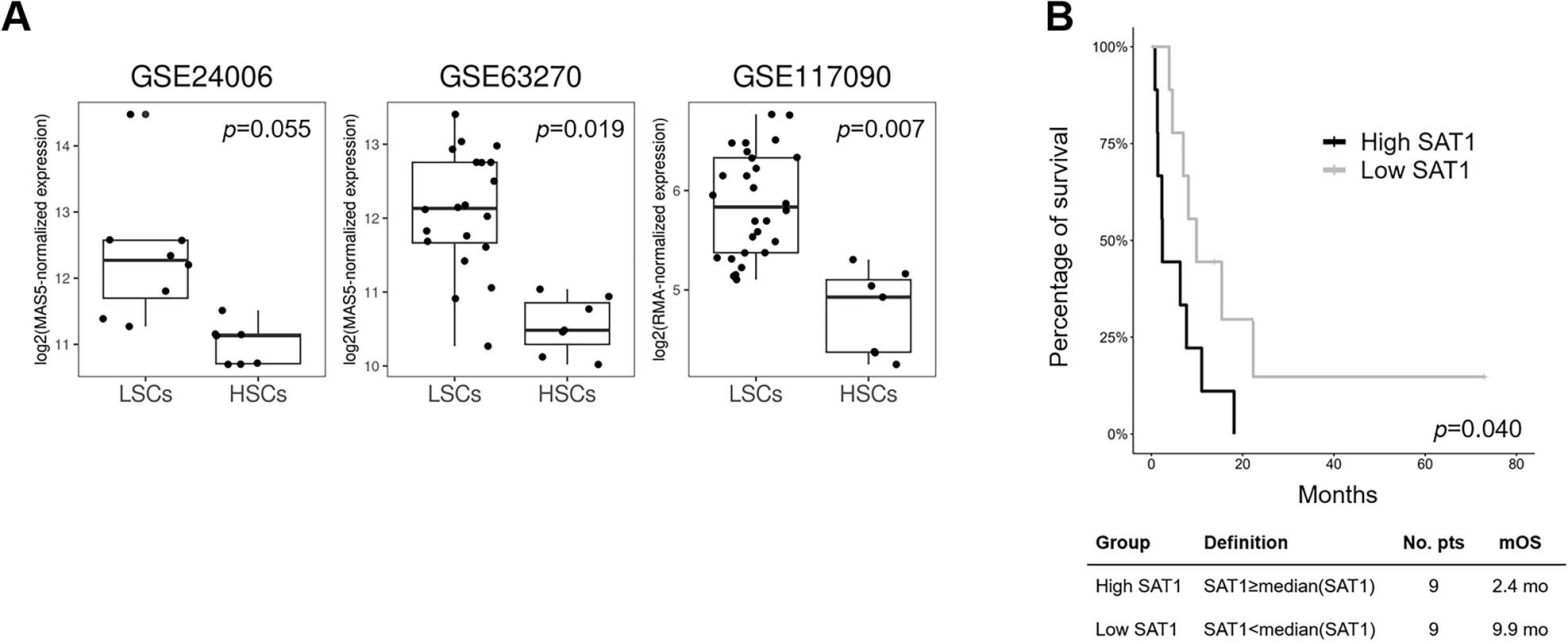
SAT1 expression and prognostic role in AML. **A** Differential Expression of *SAT1* in leukemia stem cells (LSCs) and hematopoietic stem cells (HSCs) from three independent datasets (GSE24006, GSE63270, and GSE117090) obtained from the Gene Expression Omnibus (GEO) repository. The first two datasets show log2-transformed, MAS5-normalized expression values, while the third dataset displays log2-transformed, RMA-normalized expression values. Boxplots were generated using the ggplot2 R package to visualize the distribution of SAT1 expression in the respective cell types across the different cohorts. LSC and HSC data were compared by Deseq2 and the adjusted *p*-value (Benjamini-Hochberg test) is shown in the plots. **B** Kaplan–Meyer survival curves of AML patients receiving non-intensive therapies stratified on *SAT1* transcript expression.

Several lines of evidence point towards increased SAT1 activity in AML. First, the phenotype of SAT1-transgenic mice resembled a myeloproliferative disorder, characterized by leukocytosis, with an expanded neutrophil population, an elevated number of platelets, a reduced amount of lymphocytes, and anemia compared with their wild-type littermates [21]. Bone marrow analysis revealed a decrease of common myeloid and megakaryocyte-erythroid progenitors and an expansion of granulocyte/monocyte progenitors and long-term hematopoietic stem cells (HSCs) in SAT1-transgenic compared with wild-type mice. Moreover, HSCs and multipotent progenitor cells showed an increased proliferation rate. This phenotype is indicative of enhanced myelopoiesis and accelerated differentiation of myeloid lineage cells skewed towards granulocyte/monocyte progenitor populations, along with enforced thrombocytopoiesis. Second, we recently reported that spermidine levels are reduced in AML cells isolated from the patients’ bone marrow and N1-acetylspermidine is increased in CD34^+^ AML blasts compared with their normal counterpart (CD34^+^ cord blood cells or CD33^+^ cells from the peripheral blood of healthy subjects) [9]. These results suggest a heightened SAT1 activity, in line with the phenotype of the SAT1-transgenic mouse [22]. The observed spermidine reduction looks controversial in light of the previously reported accumulation of spermidine in the mononuclear cells of AML patients [8]. However these results may partly reflect differences in the cell source (isolated cell populations [9] *versus* unselected while blood cells [8]) and the tissue type (bone marrow [9] *versus* peripheral blood [8]). Third, *SAT1* is consistently upregulated at transcript level in leukemia stem cells (LSCs) compared with HSCs (GSE63270, GSE117090 and a trend in GSE24006, https://www.ncbi.nlm.nih.gov/gds, Fig. 3A). Moreover, elevated *SAT1* transcript was associated with poor overall survival in AML patients receiving non-intensive therapies (TCGA-LAML cohort, Fig. 3B). These observations point to elevated SAT1 activity as a hallmark of AML and suggest a potential role, though not driver, of the gene in the development of myeloid neoplasia. Conversely, SAT1 exerted tumor suppressor properties in B-ALL, by triggering ferroptosis upon stress induced by reactive oxygen species (ROS) [23]. These data argue against increased SAT1 activity in ALL [8], while being in line with transcript analyses showing *SAT1* downregulation in a B-ALL cohort compared with healthy samples [23], though we cannot exclude potential differences related to post-transcriptional or post-translational mechanisms of regulation occurring at least in a subset of patients. Overall, the different role of SAT1 in AML and ALL may reflect both lineage- and differentiation stage dependencies. The role of SAT1 appears contradictory also in solid tumors. Indeed, SAT1 overexpression reduced tumor incidence in a prostate cancer model [24], while increasing it in mice predisposed to intestinal tumor formation [25]. These results may be reconciled by the spectrum of pleiotropic phenotypes mediated by elevated SAT1 levels, which include stimulation of oxidative damage, loss of polyamines and alterations of carbohydrate and lipid metabolism caused by depletion of acetyl-Coenzyme A (CoA).

In addition to biosynthetic processes occurring at intracellular level, uptake from the extracellular milieu also supports the polyamine reservoir of acute leukemia cells (Fig. 1) at least in vitro/ex vivo. AML blast showed higher uptake of a spermine-based probe compared with autologous lymphocytes [26]. Intrinsic differences were observed among AML cell lines, indicating a fine-tuning of the polyamine transport system under steady-state conditions, which may unveil a diverse sensitivity to its inhibition. An active polyamine uptake was also needed during in vitro differentiation of the AML HL-60 [27] and the murine erythroleukemia MEL cell lines [28]. Taken together, this evidence suggests that polyamines support leukemogenesis in AML and ALL, while also being required for myeloid differentiation.

## Role of polyamines in the epigenetic regulation of acute leukemias

**Fig. 4:**
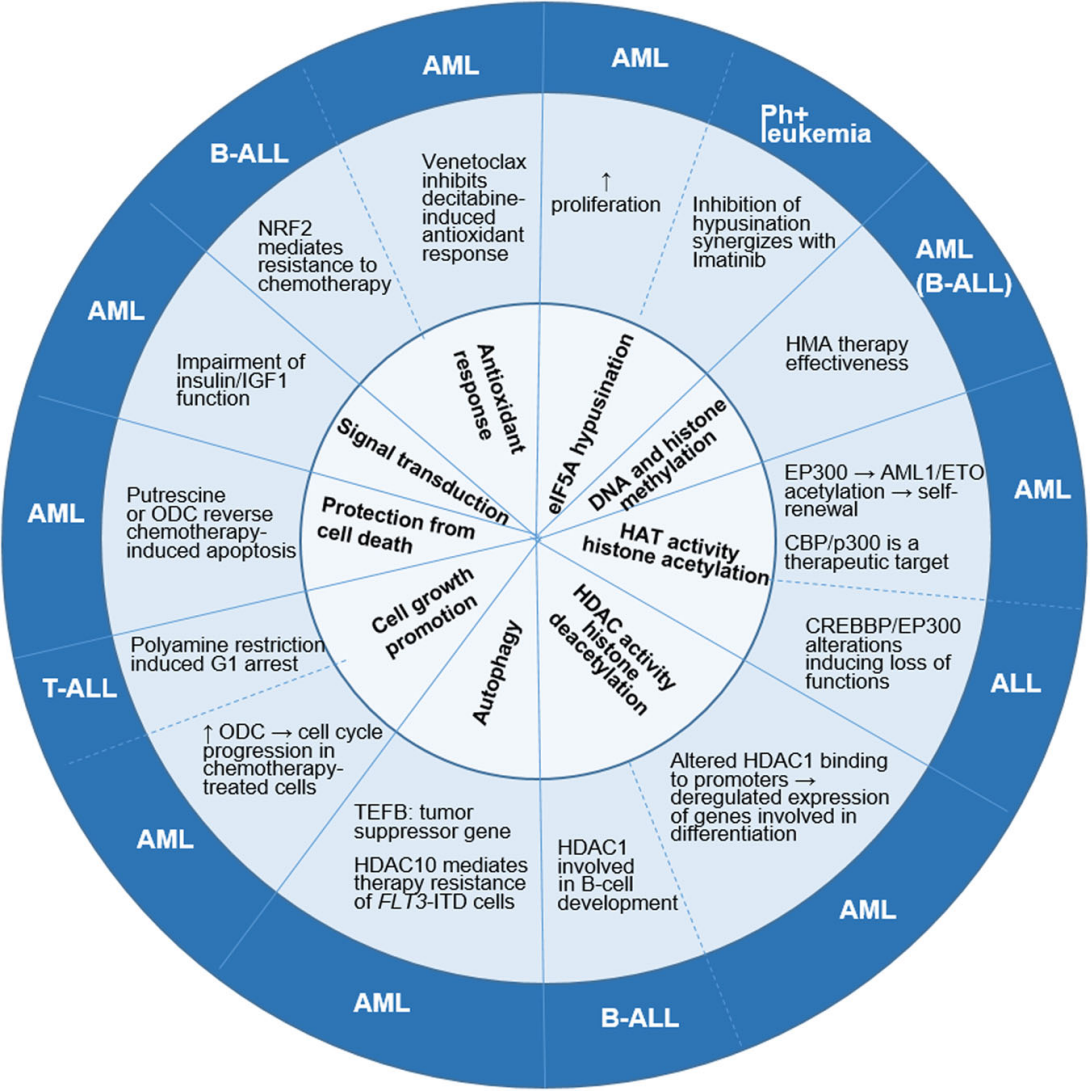
Relationship between biological processes regulated by polyamines and their pathogenic role in AML and ALL.

Recently, there is an increasing interest in understanding polyamine functions in acute leukemia, due to their linkage with epigenetic alterations (Fig. 4) that are the targets of both established and innovative therapies in the field.

### DNA methylation

SAM, which is synthesized from adenosine triphosphate (ATP) and methionine, is at the crossroad between polyamine and methionine metabolism. Moreover, it is the major donor of methyl groups for DNA and histone methylation. When polyamine requirements are elevated, a significant fraction of SAM is decarboxylated to dcSAM to support spermidine and spermine production. Therefore, heightened polyamine biosynthesis reduces SAM availability that may affect methylation processes. Bone marrow cells from mice overexpressing SAT1 showed elevated H3 methylation (trimethylated lysine K36, K9, K4 and dimethylated K79 residues) [8], along with decreased levels of spermidine and increased putrescine and N1-acetylspermidine, which may result from a feedback mechanism reducing the conversion of putrescine to spermidine when its acetylated form accumulates in the cell. As a consequence, the pool of methyl groups is replenished and the activity of histone methyltransferases can be maintained. This evidence suggests that the disruption of polyamine homeostasis contributes to the epigenetic dysregulation driving transcriptional reprogramming, which results in an altered hematopoietic cell differentiation in the SAT1-transgenic model and in AML. Accordingly, treatment of SAT1-transgenic mice with the hypomethylating agent (HMA) decitabine restored the correct proportions of neutrophils and lymphocytes in the peripheral blood and the bone marrow cells homeostasis [8]. Similarly, elevated polyamines inhibit lysine demethylase 1A (KDM1A), resulting in activation of a cancer cell stemness transcriptional program [29].

Altered methylation is a well-known pathogenic event in AML: the interaction between mutated transcription factors and epigenetic networks, as well as direct mutations in epigenetic regulators play a role in the malignant transformation of hematopoietic stem-progenitor cells (HSPCs) [30]. Accordingly, targeting DNA methylation by HMAs in combination with the BCL2 apoptosis regulator (BCL-2) inhibitor venetoclax has recently become a standard therapeutic approach in the elderly and chemotherapy-unfit AML population [31]. We can hypothesize that HMAs may partly counteract the consequences of polyamine metabolism alterations by restoring a correct methylation pattern in acute leukemias. Of note, LSCs from AML patients that were refractory or developed resistance to venetoclax/azacitidine treatment were characterized by higher spermidine and spermine levels compared with LSCs at diagnosis [32], thus opening a novel field of investigation in the post-therapy setting.

DNA hypermethylation of promoter regions is also a common epigenetic event in ALL [33–37]: some alterations are shared by all B-ALL subtypes [38], while the majority of recurrent genetic classes have unique methylation signatures [38–40]. Additionally, genome-wide DNA methylation studies of paired diagnosis-relapse ALL revealed a higher methylation of relapsed genomes [41]. Treatment of the Jurkat cell line with a spermidine analog led to downregulation of epigenetic modulators such as histone deacetylase HDAC1 and HDAC3, DNMT1 and lysine demethylases (KDM3A, KDM4B, and KDM4C) [42], suggesting a link between polyamines and methylation also in T-ALL. Indeed, decitabine demonstrated a clinical and DNA demethylation activity without severe toxicity in clinical trials on relapsed/refractory (R/R) B-ALL patients [43, 44] and in combination with venetoclax in R/R T-ALL [45–47]. Moreover, decitabine was active against *lysine methyltransferase 2A* (*KMT2A)*-positive B-cell precursor ALL models [48]. Therefore, polyamine metabolism may directly or indirectly participate in the response to HMAs in acute leukemias.

### Acetylation and deacetylation

Polyamines can modulate the activity of histone acetyltransferases (HATs), resulting in chromatin hyperacetylation. Increased activity of CREB binding protein (CREBBP), its homolog E1A binding protein P300 (EP300) and lysine acetyltransferase 2B (KAT2B) was measured in the skin of a conditional keratin6 (K6)/ODC transgenic mouse model and in tumors from ODC/RAS proto-oncogene double transgenic mice, as an indirect effect of increased polyamine levels [49, 50]. Accordingly, pharmacological depletion of polyamines reduced both HAT translation and H3/H4 acetylation in murine mammary carcinoma cells [51].

CREBBP and EP300 are frequently targeted by loss of function mutations in B/T-ALL [52–54], or rearranged with *KMT2A* or *zinc finger protein 384* (*ZNF384*) [55, 56]. *KMT2A*::*CREBBP* (or *EP300*) chimeras induced a dominant-negative loss of HAT activity, a global decrease in histone acetylation, and a higher susceptibility of leukemic cells to the histone deacetylase inhibitor [57]. In these contexts, we hypothesize that polyamines may contribute to the deregulation of the HAT wildtype allele expression and activity, when preserved.

In AML, HAT deregulation supports leukemogenesis in different genetic backgrounds [58–60]. First, EP300-mediated acetylation of the RUNX family transcription factor 1/RUNX1 partner transcriptional co-repressor 1 (AML1-ETO) fusion protein (originating from the t(8;21) translocation) is essential for the maintenance of the transforming and self-renewing properties of the oncogenic protein [60]. Second, the lysine acetyltransferase activity of CREBBP/EP300 supports leukemogenesis in different AML genetic contexts [58, 59, 61] and its pharmacological repression altered DNA replication and repair processes, induced cell cycle arrest, apoptosis (e.g. in *KMT2A*-rearranged leukemia [59]) and impairment of the clonogenic growth of primary AML cells, while sparing normal hematopoietic progenitors [58].

SAT1 function may also affect HAT activity. Indeed, SAT1-mediated acetylation of spermine/spermidine to N1-acetylspermine/N1-acetylspermidine depletes acetyl-CoA, which is also the donor of acetyl groups for histone acetylation. Therefore, SAT1 overexpression is expected to result in impaired histone acetylation. In contrast, bone marrow cells from SAT1-transgenic mice showed elevated H3K9 and H3K4 acetylation [8]. These controversial results suggest potential cell type-dependent effects or an indirect regulation exerted by putrescine, or even a compensatory mechanism arising in vivo, that remain unexplored. A genome-wide profile of both acetylated and methylated histone marks and of their combinations would clarify their role in the myeloid phenotype induced by SAT1 overexpression and also provide information on AML pathogenesis.

Bone marrow cells from SAT1-transgenic mice were also characterized by enforced HDAC1 expression compared to their wild-type counterpart [8], which appears in contrast with the heightened histone acetylation, while being in line with the increased requirements of acetyl groups to sustain N1-acetylspermine/N1-acetylspermidine production. In vivo treatment with the HDAC inhibitor trichostatin A had no effect on the myeloid expansion observed in SAT1-transgenic mice, suggesting that HDAC alterations were not drivers of the mouse phenotype. Additional evidence supports the role of polyamines in HDAC regulation. K6/ODC transgenic mice had increased HDAC activity in the skin [50], which is in line with a heightened need of acetyl groups to sustain an elevated polyamine requirement in this model. Future experiments are needed to clarify the role of polyamines in the regulation of HAT/HDAC activities in the hematopoietic lineage and in leukemogenesis.

Increased HDAC1 binding to gene promoters is a hallmark of AML and elevated HDAC1 expression has been associated with drug resistance [62]. Moreover, HDAC1 distribution presented with a specific pattern and differences across disease subtypes. HDAC1 participates in the transcriptional complex organized by the chimeric proteins encoded by *core binding factor subunit beta:: myosin heavy chain 11* (*CBFB::MYH11*) and by *RUNX1*::*RUNX1T1* on the promoters of target genes mainly involved in cell differentiation [63]. In murine erythroleukemia, HDAC1 cooperates with SPI-1 proto-oncogene (SPI1)-induced transcriptional repression by deacetylating SPI1-bound enhancers of genes involved in erythroid differentiation [64]. *HDAC1* transcript levels are also higher in pediatric ALL compared to non-leukemic cells and are significantly associated with unfavorable prognostic factors [65, 66]. Moreover, in B cells, conditional ablation of *HDAC1* and *HDAC2* hampered cell differentiation by inducing G1 arrest and apoptosis at early stage of development [67, 68].

As an additional source of complexity, evidence suggests an involvement of selected non-histone proteins in the polyamine-mediated regulation of chromatin dynamics. Polyamines inhibited the release of nucleophosmin 1 (NPM1) from chromatin in the ODC/RAS model [69], resulting in reduced dissociation of histones from the DNA. In light of the selective chromatin binding specificities of mutant NPM1 that lead to activation of oncogenic signaling in AML [70], the potential cooperation between NPM1 and polyamines in the transcriptional regulation of leukemic cells represents an interesting topic for future investigation.

## Polyamine functions relevant to the biology of acute leukemias

In addition to the regulation of the epigenome, polyamines are involved in a number of biological processes at the crossroad between cell metabolism and epigenetics in acute leukemias, with an impact on cell growth and cell death (Fig. 4). Indeed, polyamines favor cell cycle progression by regulating cyclins and cyclin-dependent kinases (CDKs), both in solid [71] and hematological tumors [72, 73]. In HL-60 cells, ODC overexpression overcame chemotherapy-mediated G1 or G2/M arrest by promoting cyclin A, D, E and CDK4 upregulation and forcing CDK1/2 activity [72]. Accordingly, in T-ALL cells, polyamine restriction induced G1 growth arrest by increasing CDKN1A/1B expression through tumor protein p53 upregulation [73]. Polyamines can also protect from cell death. Putrescine supplementation or ODC overexpression reversed chemotherapy-induced apoptosis, BCL-2 downregulation, cytochrome c release, ROS production and disruption of mitochondrial membrane potential in HL-60 cells [72]. New insights on the role of polyamines in the regulation of hypusination, autophagy, signal transduction and stress response are discussed in the following paragraphs in light of recent data that became available in the field.

### Hypusination

Hypusination is a post-translational modification limited to the eukaryotic translation inhitiation factor 5A (eIF5A), consisting in two enzymatic steps that convert a lysine residue into hypusine. Firstly, spermidine is transformed by deoxyhypusine synthase (DHS) into deoxyhypusine, that is then hydroxylated to hypusine by deoxyhypusine hydroxylase (DOHH), which interacts with the Fe(II) cofactor generated from the conversion of putrescine to spermidine. Hypusination activates eIF5A. Silencing of eIF5A2, the eIF5A factor mainly involved in cancer, sensitized AML and ALL cells to daunorubicin [74] and vincristine [75], respectively. Moreover, hypusination inhibitors reduced the proliferation of AML cell lines and synergized with the tyrosine kinase inhibitor imatinib against Ph+ leukemic cells. Of note, they were also effective against cells expressing imatinib-resistant mutations [76]. eIF5A2 activates various molecular pathways responsible for drug resistance also in solid tumors [77]. Therapeutic interventions aiding to reduce intracellular spermidine availability may prevent eIF5A from exerting its pro-tumorigenic function in acute leukemias, mainly in Ph+ ALL cases, with a potential relevance also in the 0.3% of AML cases carrying the *BCR activator of RhoGEF and GTPase::ABL proto-oncogene 1* (*BCR::ABL1*) fusion gene [78].

### Autophagy

Spermidine acts as a natural inducer of autophagy by two different mechanisms. First, it reduces the availability of acetyl-CoA, a potent inhibitor of autophagy, through acetylated polyamines production and HAT activation. Second, spermidine favors the translation of the pro-autophagic transcription factor transcription Factor EB (TFEB) through hypusinated eIF5A [79]. In AML, the MYC proto-oncogene negatively regulates the expression and function of TFEB, that exerts a tumor suppressor role through upregulation of the isocitrate dehydrogenase (IDH)1/IDH2-Tet methylcytosine dioxygenase 2 (TET2) axis, resulting in global 5-methycytosine hydroxylation, blast differentiation and death [80]. An additional factor mediating autophagy in order to protect cancer cells, (e.g. neuroblastoma cells), from chemotherapeutic drugs is HDAC10 [81]. HDAC10 also catalyzes the hydrolysis of N8-acetylspermidine to spermidine and acetate [82], thus supporting the growth of colon cancer and cervical carcinoma models under polyamine restriction [83]. We can therefore hypothesize an interplay between HDAC10 and spermidine in autophagy regulation. In AML, HDAC10 contributed to therapy resistance of *Fms-related receptor tyrosine kinase 3* (*FLT3)*-internal tandem duplication (ITD) cells [84]. Combined treatment with HDAC10 and FLT3 inhibitors (or chemotherapy) synergistically reduced the viability of *FLT3*-ITD AML models and of cells from *FLT3*-ITD relapsed patients. A potential role of polyamines and autophagy in the phenotype of *FLT3*-ITD cells has not been explored.

### Signal transduction

Polyamines regulate protein phosphorylation thanks to their ability to interact with ATP/guanosine 5′ triphosphate (GTP), which are essential mediators of signal transduction. For example, polyamines regulate the insulin receptor activity [85]. More than 80% of AML cases express insulin receptor isoform A and insulin-like growth factor 1 receptor (IGF1R), two tyrosine kinases transducing signaling through the AKT serine/threonine kinase 1 and mitogen-activated protein kinase kinase (MEK)1/2 pathways and supporting survival under serum starvation in leukemic cells [86]. Recent studies reported an insulin-resistant phenotype in AML, characterized by loss of circulating insulin in favor of glucose availability for cell growth [87]. This condition is induced by leukemia-driven production of insulin-like growth factor binding protein 1 (IGFBP1) from adipose tissue to mediate insulin sensitivity, and by loss of serotonin, microbiota-derived short-chain fatty acids and inactivation of incretin to suppress insulin secretion, resulting in an impairment of insulin/IGF1 function. Similarly, polyamines modulate estrogen receptor (ER) and epidermal growth factor receptor (EGFR) signaling pathways in human breast cancer cells through protein phosphorylation and in particular tyrosine phosphorylation of Shc adapter proteins [88], as demonstrated by ODC inhibition, that also reduced ER expression and activity [89]. Moreover, ODC silencing inhibited androgen receptor (AR) activation in prostate AR-dependent cells [90].

### Stress response

Circulating polyamines are potential substrates for oxidizing enzymes that transform them into toxic metabolites, as aldehydes and hydrogen peroxide (H_2_O_2_), leading to activation of an antioxidant response, including the increase in the nuclear levels of NFE2 like BZIP transcription factor 2 (NRF2) and the expression of detoxifying enzymes as glutathione S-transferase (GST)A1, GSTM1, NAD(P)H quinone dehydrogenase 1 (NQO1) and UDP glucuronosyltransferase family 1 member A6 (UGT1A6) through the NRF2-antioxidant response element pathway [91]. In AML, activation of NRF2/NQO1 is also mediated by *DNA methytransferase 3A* (*DNMT3A*)^R882H^ mutation [92], which impairs both the CpG methylation efficiency and the specificity of DNMT3A [93], suggesting a potential link between polyamine deregulation and altered DNMT3A activity. Moreover, treatment with venetoclax/HMA reversed decitabine-induced nuclear translocation of NRF2, expression of downstream antioxidant enzymes and BCL2-binding to NRF2/kelch-like ECH-associated protein 1 (KEAP-1) complex, resulting in an anti-leukemia activity [94]. In B-ALL, NRF2 was upregulated and activated in chemoresistant patients and its levels modulated the sensitivity of B-ALL cells to vincristine in vitro [95].

On the other hand, polyamine catabolic reactions mediated by SMOX or PAOX release H_2_O_2_ that can evoke oxidative stress and can promote ferroptosis. Ferroptosis in turn activates ODC expression through iron overload-WNT/MYC signaling, resulting in increased polyamine synthesis, thus generating a positive feedback loop in cancer cells [96]. By inducing ferroptosis, polyamine supplementation also sensitizes cancer cells or xenograft models of non-small cell lung cancer to radio/chemotherapy. These results suggest a novel targeted vulnerability mediated by elevated polyamine reservoir in cancer cells and also in acute leukemias, being MYC frequently overexpressed in both AML and ALL [97].

## Targeting polyamine metabolism for therapeutic purposes

**Fig. 5:**
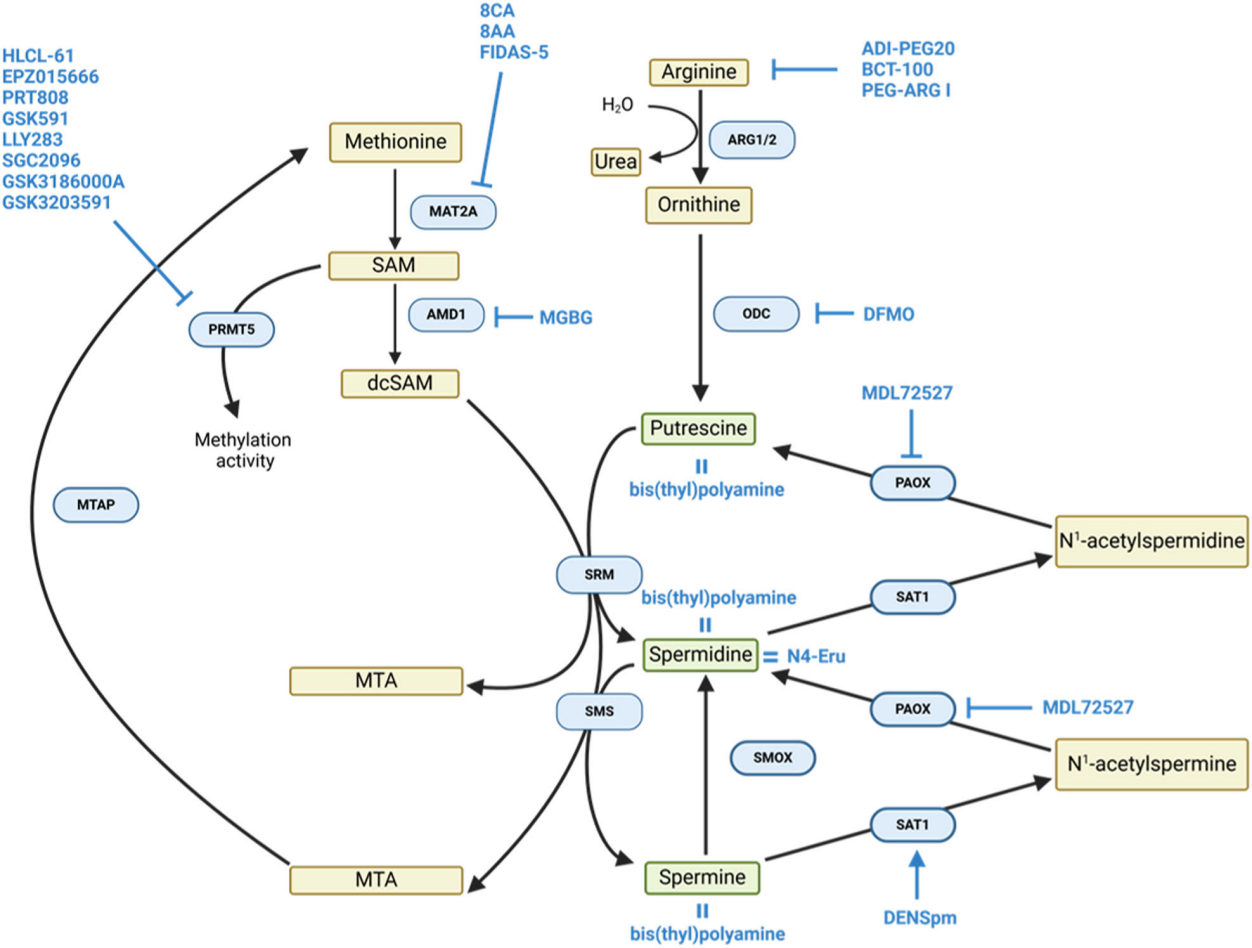
Drugs targeting polyamine metabolism and the strictly interconnected pathways that have been tested in acute leukemias (→: induction; ⏊: inhibition; =: analog).

The therapeutic potentials of inhibiting polyamine metabolism in acute leukemias has been suggested a long time ago, when a clinical response was achieved by AMD1 inhibition [98]. Additional inhibitors of polyamine biosynthesis, including spermine/spermidine analogs, the ODC inhibitor difluoromethylornithine (DFMO) and the catabolism inhibitor targeting PAOX showed a preclinical activity through reduction of cell growth and viability, or induction of blast differentiation, as summarized in Table 1 and Fig. 5. However, most of them either did not reach clinical development or did not provide beneficial effects in acute leukemia patients. Recently, some promising results have been achieved by targeting polyamine metabolism or its strictly interconnected pathways, especially in the context of selective vulnerabilities that are further discussed in the following sections.

**Table 1:** Preclinical evidence of agents targeting polyamine and strictly connected metabolic pathways in acute leukemias.

Disease	Cellular model(s)	Drug	Target	Biological effects	Reference(s)
AML	MEL	DFMO	ODC	↓ putrescine and spermidine ↑ dcSAM ↑ cell differentiation with hexamethylene bisacetamide	[127]
B-ALL	Reh	DFMO	ODC	↓ cell viability ↑ susceptibility of *RAS*-mut *vs*. wt cells	[99]
AML	HL-60	DFMO	ODC	↓ proliferation ↓ differentiation under stimuli	[128, 129]
AML	MEL	bis(thyl)polyamine analogs	analog	↓ putrescine and spermidine ↓ cell growth ↓ cell viability Hemoglobin production	[130]
T-ALL	Jurkat	N4-Eru	spermidine analog	↓ cell viability ↓ expression of genes involved in proliferation, HDACs, DNMT1, lysine demethylases Apoptosis induction ↑ expression of tumor suppressor genes	[42, 131]
T-ALL	CEM-C7-14 CEM-MycER-22	DFMO + MGBG	ODC + AMD1	Sensitization to dexamethasone	[132]
AML	MOLM-13; KG1; Kasumi-1; THP-1; KP-MO-TS	DFMO, DFMO + AMXT-1501	ODC, ODC+ polyamine uptake	↓ cell viability Apoptosis induction ↓ in vivo leukemia burden and progression (*KMT2A-*rearranged ALL models) ↑ survival of xenograft mice (*KMT2A-*rearranged ALL models)	[126]
B-ALL	PER-494; 697; RS4;11; Reh;				
T-ALL	DND-41; CEM/C1; Jurkat; Loucy; P12-Ichikawa; KE-37; HBP-ALL; MOLT-4; SUP-T1; RPMI-8402				
MPAL	PER-703; PER-485				
AML	THP1	MDL72527	PAOX	↓ polyamines ↑ N1-acetylspermidine Sensitivity to doxorubicin	[133]
AML	Human LSC	DENSpm	SAT1 induction	↓ putrescine and spermidine ↓ cell viability ↓ colony forming ability ↓ in vivo engraftment and leukemia burden ↓ protein synthesis and eIF5A hypusination	[100]
AML	Primary human leukemic cells	ADI-PEG 20	arginine	↓ arginine Apoptosis induction in ASS1-deficient AML ↓ leukemia burden in PDX mice Effective combination with cytarabine in vivo	[105]
B-ALL	REH; TOM1; NALM-6	BCT-100	arginine	↓ arginine ↓ cell viability ↓ in vivo engraftment Synergism with dexamethasone	[102]
T-ALL	Jurkat; MOLT-4Primary human leukemic cells				
T-ALL	CCRF-CEM; MOLT-4; MOLT-3; Jurkat; H9; Loucy; HPB-ALL; KOPTK1; T-ALL-1; ALL-Sil	peg-Arg I	arginine	↓ cell growth Apoptosis induction Protein synthesis arrest ↑ survival of xenograft mice in combination with cytarabine	[103, 104]
AML	MOLM-13; OCI-AML3; MV4-11 Primary human leukemic cells MLL-AF9- transformed primary mouse cells	FIDAS-5	MAT2A	↓ cell growth ↓ colony forming ability ↑ apoptosis ↓ H3K36me3 active elongation mark	[109]
AML	MV4-11; MOLM‐14, OCI‐AML3; MOLM‐13; KG-1a Primary human leukemic cells	8CA, 8AA	MAT2A (among others)	↑ apoptosis ↑ survival of xenograft mice Synergism with quizartinib Synergism with venetoclax	[107, 110, 111]
AML	MV4-11; THP1 FLT3-ITD primary human leukemic cells FLT3-wt primary human leukemic cells	HLCL-61	PRMT5	↓ cell viability Apoptosis induction Cell differentiation	[114]
AML	MV4-11; MOLM-13 FLT3-ITD primary cells	EPZ015666, PRT808	PRMT5	Synergism with FLT3 inhibition	[115]
AML	OCI-AML-20 *SRSF2*-mut primary cells *EVI1*-high primary cells	GSK591, LLY283, SGC2096	PRMT5	Oxidative stress ↓ cell growth Senescence induction	[116]
AML	MV4-11; OCI-AML3; SKNO-1; THP-1	GSK3186000A	PRMT5	Synergism with PARP inhibition	[117]
AML	Splicing factors-mut primary cells	GSK3203591	PRMT5	Apoptosis induction	[118]
AML	*srsf2*^P95H^ *MLL-AF9* THP1; MOLM13 *SRSF2*^P95L^ iPSCs Splicing factors-mut primary cells	EPZ015666+ MS023	PRMT5+ type I PRMTs	↓ cell viability ↑ survival of xenograft and PDX mice	[118]
AML	*MLL-ENL/Nras*^G12D^ *MLL*-*AF9* MOLM-13	EPZ015666	PRMT5	↓ cell growth Cell differentiation ↑ survival of xenograft mice	[119]
B-ALL T-ALL	NALM6 Primary cells	HLCL-61	PRMT5	Apoptosis induction Cell differentiation	[120]

*iPSCs* induced pluripotent stem cells, *LSC* leukemia stem cells, mut mutated, *PDX* patient-derived xenograft, *vs* versus, *wt* wildtype.

### Inhibition of polyamine biosynthesis

In B-ALL, *KRAS* mutation induced a selective vulnerability to DFMO [99] by forcing polyamines and proline biosynthesis at the expenses of methionine and arginine. Accordingly, *KRAS*^G12D^ mutant Reh B-ALL cells showed reduced intracellular and extracellular levels of methionine and accumulation of MTA in the culture medium compared with wildtype cells, due to KRAS-mediated activation of the AKT/mechanistic target of rapamycin kinase (mTOR) signalling that induced methionine catabolism through AMD1.

### Modulation of polyamine catabolism

AML cells were sensitive to the polyamine analogueN(1),N(11)-diethylnorspermine (DENSpm) that forced SAT1 activity leading to spermidine and spermine acetylation followed by cellular export [100]. DENSpm treatment reduced LSC viability, colony forming ability and engraftment in immune-deficient mice, and also delayed disease progression in vivo. At functional level, eIF5A hypusination and KAT7 protein level were altered by the drug. These results may appear in contrast with the data from SAT1-transgenic mice and from AML public cohorts, arguing for a leukemia-supporting role of SAT1. We can therefore hypothesize a threshold effect for SAT1 activity and/or the ratio of polyamines/acetylated polyaminse in AML cells, shifting the balance from an oncogenic to a tumor suppressor signal, also in relationship with the genetic background. Future studies are needed to clarify the role of SAT1 in AML.

### Therapeutic targeting of arginine auxotrophy

Alternative approaches to deplete the polyamine pool involve the disruption of upstream or converging metabolic pathways and in particular arginine supply. Our metabolomics data showed reduced arginine levels in AML blasts compared with their normal counterpart [9], suggestive of high or rapid consumption. Conversely, it was abundant in LSC from AML patients compared with HSPCs [100]. Unlike non-malignant cells, which can regenerate arginine from alternative amino acids, and in particular from citrulline through the arginosuccinate synthase (ASS) - arginosuccinate lyase (ASL) axis, arginine auxotrophy is a common characteristic of AML [101] and ALL [102] blasts, thus also representing a potential vulnerability. In B/T-ALL, arginine depletion by the pegylated human recombinant arginase BCT-100 reduced leukemia engraftment, prolonged survival in xenograft models and synergized with dexamethasone [102]. Moreover, pegylated arginase I (peg-Arg I) was effective against T-ALL [103] also in combination with cytarabine, by inducing protein synthesis arrest and phosphorylation of eIF2α, which mediated apoptosis [104]. Accordingly, ex vivo culture of AML blasts under arginine-depleted conditions resulted in G0/G1 cell cycle arrest and cell death [105]. Moreover, ASS-deficient AML blasts were notably sensitive to pegylated arginine deiminase (ADI-PEG 20), a therapeutic arginine-degrading enzyme derived from *Mycoplasma sp* [105]. However, the combination of ADI-PEG 20 and cytarabine did not improve the survival of patients compared to cytarabine monotherapy [106]. A better understanding of the molecular and metabolic mechanisms that hampers the clinical efficacy of these agents may guide the optimization of combination therapies towards improved activity.

### Strategies targeting the methionine-polyamine homeostasis and the leukemic cell epigenome

Another metabolic pathway strictly interconnected with polyamines involves methionine, whose levels are significantly reduced by venetoclax in AML blasts [107]. In particular, MAT2A and PRMT5 are emerging therapeutic targets at the crossroad between methionine/polyamine metabolism and epigenetic regulation.

MAT2A is a negative prognostic marker [107] and a selective vulnerability in AML [108]. Its pharmacological inhibition by FIDAS-5 was suggested to specifically reduce the active elongation mark H3K36me3 [109]. MAT2A impairment was also induced by the 8-chloro-adenosine (8CA) and 8-amino-adenosine (8AA) nucleoside analogs that inhibited RNA splicing by reducing methyltransferase 16, RNA N6-adenosine (METTL16) protein [107]. Of note, 8CA, that showed an efficacy against AML models as single agent [110], synergized with venetoclax [111] both in vitro against LSC and in vivo in patient-derived xenograft (PDX) models. Its activity was mediated by inhibition of the methionine-MAT2A-SAM axis, thus resulting in impaired polyamine biosynthesis due to substrate deficiency and global reduction of histone methylation with no changes in DNA/RNA methylation [107]. In a phase I trial, 8CA monotherapy induced a transient peripheral blood cytoreduction, suggesting that combination therapies are required to achieve significant clinical responses.

SAM is a substrate for both polyamine biosynthesis through AMD1, and methylation reactions through PRMT5 that is in turn inhibited by MTA. It has been demonstrated that MTAP deficiency, leading to MTA accumulation, offers a therapeutic window by creating a hypomorphic PRMT5 state that selectively sensitizes cancer cells to PRMT5 inhibition [112]. These findings are relevant to T-ALL, since *MTAP* deletion is a recurrent event and sensitized T-ALL CEM cells to methionine deprivation [113].

PRMT5 inhibition is a selective vulnerability also in some AML subtypes. PRMT5-mediated H4R3 dimethylation increased expression of SP1, leading to transcriptional activation of FLT3, which was reversed by the HLCL-61 PRMT5 inhibitor [114]. The treatment induced apoptosis and forced myeloid differentiation of both *FLT3*-ITD and wildtype cells. Moreover, EPZ015666- or PRT808-mediated targeting of PRMT5 synergized with the FLT3 inhibitor gilteritinib in *FLT3*-ITD models [115]. In MDS1 and EVI1 complex locus (EVI1)-overexpressing leukemia, PRMT5 inhibition decreased the levels of the spliced cytoplasmic form of activating transcription factor 4 (ATF4) protein, resulting in increased oxidative stress, growth arrest and senescence [116]. PRMT5 inhibition also affected the splicing of the multifunctional histone-modifying and DNA-repair factor KAT5, resulting in alterations of its lysine acetyltransferase activity and in the consequent impairment of homologous recombination, that sensitized cells to poly(ADP-ribose) polymerase (PARP) inhibitors [117]. Moreover, leukemias carrying splicing factors mutations, that are intrinsically sensitive to splicing perturbations, responded to the selective PRMT5 inhibitors GSK3203591 and EPZ015666, and to their combination with the type I PRMT inhibitor MS023 both in vitro and in vivo, as confirmed by the prolonged survival of mice transplanted with *serine and arginine rich splicing factor 2* (*srsf2)*^P95H^*MLL-MLLT3 super elongation complex subunit (MLLT3)*-transformed leukemia [118]. The drug combination altered splicing events resulting in increased DNA damage and cell cycle arrest. In *KMT2A*-rearranged AML, PRMT5 expression is part of the transcriptional program driven by the co-activator polymerase-associated factor complex that binds the fusion protein. PRMT5 inhibition by EPZ015666 was able to override the differentiation block induced by *KMT2A* chimeras through transcriptional silencing of CDKN1A and to delay disease progression and increase survival of *MLL-MLLT1/Nras*^G12D^ mice [119]. PRMT5 silencing also induced differentiation from the pre-B to immature B stage in B-ALL cells from pediatric patients [120].

The preclinical data paved the way for a phase 1 study of the PRT543 inhibitor of PRMT5, that was open to R/R AML patients (#NCT03886831; https://clinicaltrials.gov/). PRT543 treatment was well tolerated and inhibited target engagement and functional activity in myelodysplastic syndrome or myelofibrosis patients, with reduction of inflammatory markers and improved symptoms in selected cases. Although no data is currently available on the AML cohort, these results suggest that PRMT5 inhibition can be of clinical utility in selected patients subgroups.

## Conclusions

Fifty years of research on polyamine metabolism have generated evidence of its role in malignant cells, especially in solid tumors. The results here discussed show that intracellular polyamine concentrations regulate multiple cellular functions also relevant to leukemogenesis, including cell viability, proliferation and differentiation. In particular, the fine interplay between alterations of polyamine metabolism or its related pathways and epigenetic regulation of leukemic cells through methylation and acetylation processes may be involved in the maintenance of leukemogenic transcriptional programs, in the regulation of cell differentiation and in the response to HMAs. How polyamines regulate the epigenome in leukemias, especially during treatment remains an open question, along with their functional role in therapy resistance. Intrinsic differences exist between AML and ALL, likely due to specific polyamine requirements according to the differentiation stage and lineage commitment as observed for SAT1, that deserve future investigation. Overall, most results are in line with the observations from solid tumors, including some controversial ones. The information reviewed here can inspire novel research directions in the acute leukemia field that can benefit of the recent technological advancements allowing the integration of global genetic, epigenetic, transcriptomic and metabolic information. Moreover, driver genes, as RAS, FLT3, NPM1, EVI1 and KMT2A shape the interaction between polyamine metabolic pathway and leukemia-supporting functions, resulting in selective vulnerabilities, as unveiled by drugs targeting PRMT5. While the interaction between polyamine metabolism and pan-cancer drivers including MYC, the RAS/RAF/MEK and the PI3K/AKT/mTOR pathways or tumor suppressors as p53 are established, future studies should address the interplay between the leukemia-specific molecular alterations and polyamine metabolism, aiding to biomarker-driven research for clinical translation.

So far, the clinical benefit of polyamine-targeting agents has been limited due to compensatory mechanisms allowing the malignant cells to refill the polyamine pool, for example by increased uptake from the microenvironment through the transport system when biosynthesis inhibitors (as DFMO) are administered as monotherapy [121]. Moreover, dose-limiting adverse events, including ototoxicity, gastrointestinal toxicity and neurological symptoms have hampered the clinical development of the early generation inhibitors [122–125]. Current efforts are focused on the identification of therapeutic combinations able to achieve clinical efficacy at low and tolerable drug doses. Recently, promising preclinical results were obtained by combined inhibition of polyamine biosynthesis and import in acute leukemias [100, 126]. However, cardiac toxicity events induced by the drug combination have been reported in a clinical trial on pediatric patients affected by high-risk neuroblastoma and diffuse intrinsic pontine glioma (https://www.neuroblastoma.org.au/dfmo-combination-therapy). Therefore, in the next years, the major challenge that the research on polyamine metabolism in leukemia will have to face is represented by the translation of the recent findings into valuable therapeutic opportunities, including the identification of the best therapeutic combinations balancing clinical utility and tolerability. The evidence discussed in this review provides the rationale for a deepen investigation of polyamine metabolism in acute leukemias and its druggability, aiding to an informed targeting of metabolic vulnerabilities in the context of novel clinically valuable therapeutic combinations.

## Data Availability

The datasets analyzed during the current study are publicly available in the cBioPortal for Cancer Genomics and Gene Expression Omnibus (GEO) repositories.
